# Temperature‐Dependent Separation of CO_2_ from Light Hydrocarbons in a Porous Self‐Assembly of Vertexes Sharing Octahedra

**DOI:** 10.1002/advs.202308028

**Published:** 2024-02-02

**Authors:** Shun Li, Qing Li, Ting Chen, Zhen‐Yu Ji, Guo‐Ling Li, Ming‐Yan Wu, Li‐Yi Meng, Zi‐Ang Nan, Wei Wang, Zhu Zhuo, Fengru Fan, You‐Gui Huang

**Affiliations:** ^1^ CAS Key Laboratory of Design and Assembly of Functional Nanostructures and Fujian Provincial Key Laboratory of Nanomaterials Fujian Institute of Research on the Structure of Matter Chinese Academy of Sciences Fuzhou Fujian 350002 China; ^2^ Xiamen Key Laboratory of Rare Earth Photoelectric Functional Materials Xiamen Institute of Rare Earth Materials Haixi Institutes Chinese Academy of Sciences Xiamen Fujian 361021 China; ^3^ State Key Laboratory of Structure Chemistry Fujian Institute of Research on the Structure of Matter Chinese Academy of Sciences Fuzhou Fujian 350002 China; ^4^ State Key Laboratory of Physical Chemistry of Solid Surfaces iChEM College of Chemistry and Chemical Engineering Innovation Laboratory for Sciences and Technologies of Energy Materials of Fujian Province (IKKEM) Xiamen University Xiamen 361005 China

**Keywords:** anion coordination, CO_2_/C_2_H_2_ separation, diffusion regulation, octahedra, supramolecular assembly

## Abstract

Design of flexible porous materials where the diffusion of guest molecules is regulated by the dynamics of contracted pore aperture is challenging. Here, a flexible porous self‐assembly consisting of 1D channels with dynamic bottleneck gates is reported. The dynamic pendant naphthimidazolylmethyl moieties at the channel necks provide kinetic gate function, that enables unusual adsorption for light hydrocarbons. The adsorption for CO_2_ is mainly dominated by thermodynamics with the uptakes decreasing with increasing temperature, whereas the adsorptions for larger hydrocarbons are controlled by both thermodynamics and kinetics resulting in an uptake maximum at a temperature threshold. Such an unusual adsorption enables temperature‐dependent separation of CO_2_ from the corresponding hydrocarbons.

## Graphical abstract

A porous flexible supramolecular material that is composed of vertexes sharing octahedra is assembled from dimeric [Mn_2_L_2_Cl_2_]^2+^ bearing pendant naphthimidazolylmethyl arms. The self‐assembly is characteristic of the channels with small dynamic gates. The local dynamics of the pendant naphthimidazolylmethyl arms leads to unusual diffusion‐regulatory adsorption of light hydrocarbons, thus leading to temperature‐dependent separation of CO_2_ from the corresponding hydrocarbons.

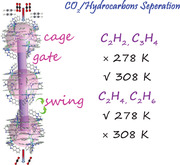

## 1 Introduction

External stimuli‐induced structural multi‐stability is regarded as one of the important features of flexible porous frameworks,^[^ ^1^, ^2^, ^3^ ^]^ and such thermodynamically controlled framework flexibility may give rise to mixed‐gas separation through a gating mechanism.^[^ ^4^, ^5^, ^6^ ^]^ On the other hand, some kinetically controlled flexible frameworks may adsorb oversize guest molecules by transiently opening their small apertures.^[^ ^7^, ^8^, ^9^, ^10^, ^11^, ^12^ ^]^ Guest diffusion process in these kinetically controlled flexible frameworks may be regulated by temperature, thus leading to substantial temperature‐responsive adsorption.^[^ ^13^ ^]^ Specifically, the guest‐diffusion is strongly impeded at low temperature, whereas diffusion is facilitated at elevated temperatures. Indeed, some kinetically controlled flexible frameworks have been explored to control the diffusion of guest species, and highly selective oxygen/argon, ethylene/ethane separation,^[^ ^14^ ^]^ and water iso‐topologues separation^[^ ^15^ ^]^ have been achieved.

Guest diffusion in diffusion‐regulatory frameworks involves interconversion between the transient gate‐opening state and the thermodynamically stable gate‐closed state.^[^ ^16^, ^17^, ^18^, ^19^ ^]^ Such interconversion can arise from dynamic mechanisms such as framework distortion, internetwork displacement, and rotation/swing of non‐coordinated groups.^[^ ^20^, ^21^, ^22^, ^23^, ^24^, ^25^ ^]^ Among these dynamic behaviors, framework distortion and internetwork displacement, characteristic of global framework flexibility, can easily induce permanent structural transformation leading to multi‐stability under different conditions.^[^ ^26^, ^27^, ^28^, ^29^ ^]^ On the other hand, local molecular motions, such as the rotation/swing of uncoordinated functional groups, induce momentary structural variations.^[^ ^13^, ^30^ ^]^ However, the aperture variations induced by such local dynamic motions are usually too small to be used for controlling guest diffusion. Therefore, the rational design of diffusion‐regulatory dynamic porous materials remains an ongoing challenge.

**Figure 1:**
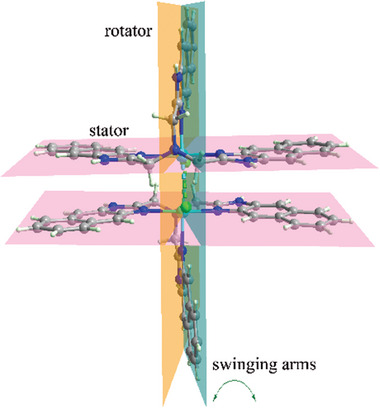
The dimeric [Mn_2_L_2_Cl_2_]^2+^ building block of **1**. The thermal swing of the two pendant naphthimidazolylmethyl arms is expected to control the gate size of the channels in **1**.

Pendant functional groups on ligands have proven effective for regulating guest diffusion.^[^ ^14^, ^15^ ^]^ The apertures of dynamic frameworks can be continuously tuned from closed to open by the cooperative motions of pendant groups, like the IRIS stop used in optical cameras. For example, the swing of the ethyl groups on 3,5‐diethyl‐1,2,4‐triazole (Hetz)^[^ ^18^ ^]^ and the flip‐flop motion of phenothiazine‐5,5‐dioxide (OPTz) on ligand OPTz‐ipa which comprises isophthalic acid and phenothiazine‐5,5‐dioxide were used to regulate gas diffusion.^[^ ^14^ ^]^ Herein, we report a porous supramolecular self‐assembly {[(Mn_2_L_2_Cl_2_)Cl_2_]·2(btcn)}_n_ (**1**) constructed by [Mn_2_L_2_Cl_2_]^2+^, Cl^‒^, and btcn via the cooperation of hydrogen bonding (anion coordination)^[^ ^31^ ^]^ and *π*–*π* stacking interaction (btcn = 1,3,5‐benzenetricarbonitrile). The [Mn_2_L_2_Cl_2_]^2+^ ion is cross‐shaped, in which the flat moiety may act as a stator and the two pendant arms may act as rotators (**Figure** **1**). The porous self‐assembly consists of 1D channels with small apertures defined by three pendant naphthimidazolylmethyl arms. The dynamics of the pendant naphthimidazolylmethyl arms enables the sorption of oversized guest molecules, and thus temperature‐dependent separation of CO_2_/C_2_H_2_.

## 2 Crystal Structure

**Figure 2:**
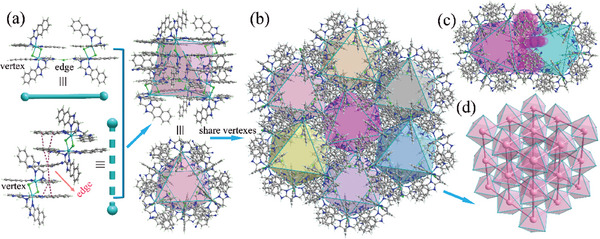
a) The scheme showing six [Mn_2_L_2_Cl_2_]^2+^ are connected by six Cl^‒^ and six btcn molecules forming an octahedron. b) The view of each octahedron sharing the vertexes with its six neighbors. c) The junction part of two adjacent octahedrons, the shared [Mn_2_L_2_Cl_2_]^2+^ vertex is shown in space‐filling mode. d) The simplified **pcu** network of **1** if considering the octahedrons as nodes.

**Figure 3:**
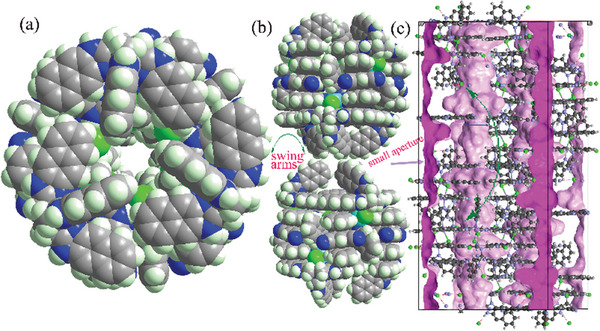
a) Top view of the 1D channel in **1**. b) Cross‐section view of the small aperture connecting two adjacent octahedral cages. c) Side view of the 1D channel, the inner and outer surfaces of the channel are shown in purple and pink, respectively, the green arrow indicates the diffusion pathway and the diffusion is regulated by the dynamic aperture.

The reaction of MnCl_2_·4H_2_O, L, and btcn (Figure S1, Supporting Information) in the mixture of MeOH and acetone (2:10, v/v) yield single crystals of {[(Mn_2_L_2_Cl_2_)Cl_2_]·2(btcn)}_n_ (**1**), which is formed by the self‐assembly of dimeric [Mn_2_L_2_Cl_2_]^2+^ ions, Cl^‐−^, and btcn as revealed by the High‐Resolution Mass Spectrometry (HR‐MS) analysis (Figure S2, Supporting Information). **1** crystallizes in the trigonal space group *R–3* (CCDC No. 2300877)^[^ ^32^ ^]^ (Table S1, Supporting Information) with the asymmetric unit containing a [Mn_2_L_2_Cl_2_]^2+^ ion, two Cl^‐−^ anions, and two btcn molecules. The [Mn_2_L_2_Cl_2_]^2+^ ion is formed by two [MnLCl]^+^ monomers through two weak Mn─Cl bonds (bond length: ≈2.765 Å). The dihedral angle between the two pendant naphthimidazolylmethyl arms is ≈16.2° (Figure 1). These [Mn_2_L_2_Cl_2_]^2+^ ions hydrogen bond to Cl^−^ forming a 3D framework, in which the cavities in the wall were tessellated by btcn molecules through host‒guest *π*–*π* interactions. Attempts to obtain isostructural framework without btcn molecules were not successful, implying btcn molecules are crucial to stabilize the structure. The hierarchical structural complexity of the framework is best appreciated by describing it in a bottom‐up fashion. Six [Mn_2_L_2_Cl_2_]^2+^ ions are connected by six Cl^−^ anions and six btcn molecules through hydrogen bonds and *π*–*π* interactions, respectively, forming an octahedron (**Figure** **2**a). The octahedron possesses two small triangular gates which are defined by three pendant naphthimidazolylmethyl arms. The vertexes of the octahedron are occupied by [Mn_2_L_2_Cl_2_]^2+^ ions, and Cl^−^ anions and btcn molecules located on the edges. Each such octahedron connects to its six neighbors by sharing a [Mn_2_L_2_Cl_2_]^2+^ ion with its each neighbor (Figure 2b,c), leading to the intricate framework structure. From a topological viewpoint, if each octahedron is considered as a six‐connected node, the framework structure of **1** can be described as a **pcu** network (Figure 2d).^[^ ^33^ ^]^ In the framework, octahedrons are orderly aligned along the *c*‐axis giving rise to 1D channels (**Figure** **3**). The wall of the channel is formed by hexagonally aligned *π*‐stacked helical columns (Figure S3, Supporting Information), and the void of the channels is formed by the cavities within the octahedrons that are connected by their small gates (Figure 3). The dynamics of these small gates may control the diffusion of guest molecules in the channels. As determined by PLATON software package,^[^ ^34^ ^]^ the total void volume (*V*_void_) is 8773.4 Å^3^ per unit cell, which is 21.0% of the unit volume.

## 3 Stabilities and Regeneration

Thermogravimetric (TG) analysis (Figure S4, Supporting Information) with the sample heated under an argon stream domonstrated **1** is stable beyond 250 °C as confirmed by powder X‐ray diffraction (PXRD) (Figure S5, Supporting Information) and single‐crystal X‐ray diffraction (SCXRD) analyses. The mass‐loss process below 100 °C is attributed to the removal of solvent molecules. The acid stability and regeneration property of **1** were also investigated. As we can see, **1** is robust after immersion in an acidic aqueous solution (pH 2) for 24 h (Figure S5, Supporting Information). Also, **1** can be readily recovered by recrystallization (Figures S5 and S6, Supporting Information). In specific, dissolving the crystals of **1** in the MeOH/acetone mixture (2:10, v/v) gives rise to a clear colorless solution. Upon evaporating the solvent at room temperature, crystals of **1** are regenerated (Figure S6, Supporting Information).

## 4 Adsorption Properties

### 4.1 Porosity Evaluation

The exceptional stability of **1** promoted us to evaluate its permanent porosity. The initial result is rather surprising, no porosity is observed when conducting the N_2_ (diameter: 3.64 Å) and Ar_2_ (diameter: 3.4 Å) adsorption experiments on activated**‐1** at 77 and 87 K, respectively (Figure S7, Supporting Information). We assume this phenomenon may originate from the weak N_2_/Ar_2_‒framework affinities and/or the small apertures that prohibit N_2_/Ar_2_ molecules to diffuse through.^[^ ^35^, ^36^, ^37^ ^]^ Therefore, we collect the sorption isotherm of CO_2_ (diameter: 3.3 Å) at 195 K, a smaller probe molecule. The result reveals a stepwise isotherm with an uptake of 120.8 cm^3^ (STP) g^−1^ at 1 *P*/*P_o_* = 1.0 (Figure S7, Supporting Information), and the notable step near *P*/*P_o_* = 0.4 implies a possible structural transformation. The evaluated Brunauer–Emmett–Teller (BET) surface area and pore volume are 262 m^2^ g^−1^ (calculated based on the adsorption data in the relative pressure of 0.05‒0.30) and 0.19 cm^3^ g^−1^, respectively, with a narrow pore‐size distribution near the average pore diameter of 6.11 Å (Figure S7, Supporting Information). The evaluated pore volume is consistent with the theory value of 0.23 cm^3^ g^−1^ obtained from single crystal structure, and the BET surface area is comparable to some reported MOFs and hydrogen‐bonded organic frameworks (HOFs).^[^ ^38^, ^39^, ^40^, ^41^, ^42^, ^43^ ^]^

### 4.2 Adsorption Isotherms

**Figure 4:**
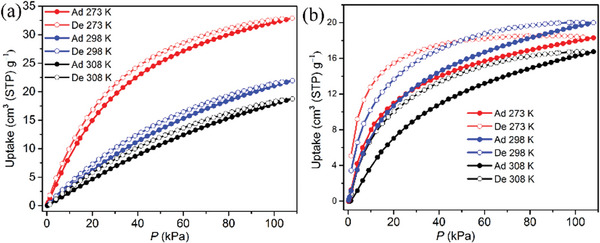
The CO_2_ a) and C_2_H_2_ b) sorption isotherms of activated‐**1** at 273, 298, and 308 K.

As revealed by temperature variable SCXRD analyses, no obvious structural variation occurred along the temperature except that the size of the triangle gates is temperature responsive due to the thermal swing of the pendant naphthimidazolylmethyl arms. The size gradually enlarges from ≈3.8 to ≈4.2 Å (considering van der Waals radius of atoms) from 100 to 308 K (Figure S8, Supporting Information). The larger gates at higher temperatures may facilitate the diffusion of oversized guest molecules in the channels.^[^ ^14^, ^15^, ^18^ ^]^ To a certain oversize molecule, the diffusion rate may significantly increase at a certain temperature thus leading to adsorption controlled by both thermodynamics and kinetics. To verify our hypothesis, the sorption isotherms of CO_2_ and various light hydrocarbons (ethane, ethylene, acetylene, propylene, and propyne) were measured at 273, 298, and 308 K on ASAP 2020 (The exposure times are automatically set to be relatively shorter compared to those of 3Flex) (**Figure** **4**; Figure S9, Supporting Information). With decreasing temperature, the CO_2_ uptake increases from 18.74 cm^3^ (STP) g^‒1^ at 308 K and 108 kPa to 32.89 cm^3^ (STP) g^‒1^ at 273 K and 108 kPa with an isosteric heat of adsorption (*Q*_st_) at zero coverage of 34.8 kJ mol^‒1^ (**Figure** **4**a; Figure S10, Supporting Information). The diameter of CO_2_^[^ ^35^ ^]^ is smaller than the size of the aperture. Therefore, CO_2_ molecules can readily diffuse through the apertures without obvious hindrance resulting in such thermodynamically dominant sorption isotherms. In contrast, the sorption isotherms for light hydrocarbons are very unusual (Figure 4b; Figure S9, Supporting Information). Adsorption for all the measured hydrocarbons cannot reach equilibrium, thus all the isotherms show large hysteresis which could be attributed to kinetic restrictions.^[^ ^44^ ^]^ At a certain pressure, the uptake does not increase with decreasing temperatures. For example, at 108 kPa the acetylene (C_2_H_2_) uptake of 20.00 cm^3^ (STP) g^‒1^ at 298 K is the highest among the three measured temperatures (Figure 4b). In addition, the sorption isotherms for propylene and propyne were also measured on 3Flex at 298 K (The exposure times were automatically set to be much longer compared to those on ASAP 2020.; Figure S11, Supporting Information). The uptakes of both of the hydrocarbons were significantly improved when the exposure times were prolonged, which is characteristic of diffusion‐regulatory porous materials (Figure S12, Supporting Information).^[^ ^14^, ^15^, ^45^, ^46^, ^47^ ^]^

No significant structural transformation of activated‐**1** can be observed from 100 to 308 K as revealed by temperature variable SCXRD analyses, thus the observed hydrocarbons adsorption abnormality is probably dominated by kinetic factors, other than other possible factors such as thermal breathing and phase transitions.^[^ ^48^, ^49^, ^50^ ^]^ The apertures slightly enlarge (Figure S8, Supporting Information) and the thermal swing of the pendant naphthimidazolylmethyl arms can be enhanced with increasing temperatures, thus the diffusion rates of these oversize hydrocarbons increase leading to unusual adsorption. To evaluate the diffusion rates of CO_2_ and C_2_H_2_ in the channels of activated‐**1**, the CO_2_‐adsorbed and C_2_H_2_‐adsorbed structures of activated‐**1** (Figure S13, Supporting Information) were simulated by Grand Canonical Monte Carlo (GCMC)^[^ ^51^ ^]^ and the mean square displacements (MSD) of C_2_H_2_ in activated‐**1** were analyzed by the molecular dynamics (MD) method.^[^ ^51^ ^]^ The diffusion rates of C_2_H_2_ at 273, 298, and 308 K were calculated to be 1.812 × 10^‒6^, 4.215 × 10^‒6^, and 4.749 × 10^‒6^ cm^2^ s^−1^, respectively. Compared to the slight increase of diffusion rate from 298 to 308 K, the increase from 273 to 298 K is much more significant. Therefore, both the two conflict factors of gas‐framework affinity and diffusion limitation influence the adsorption behavior of C_2_H_2_ and the C_2_H_2_ uptake unusually increases from 273 to 298 K.

### 4.3 Gases Separations

**Figure 5:**
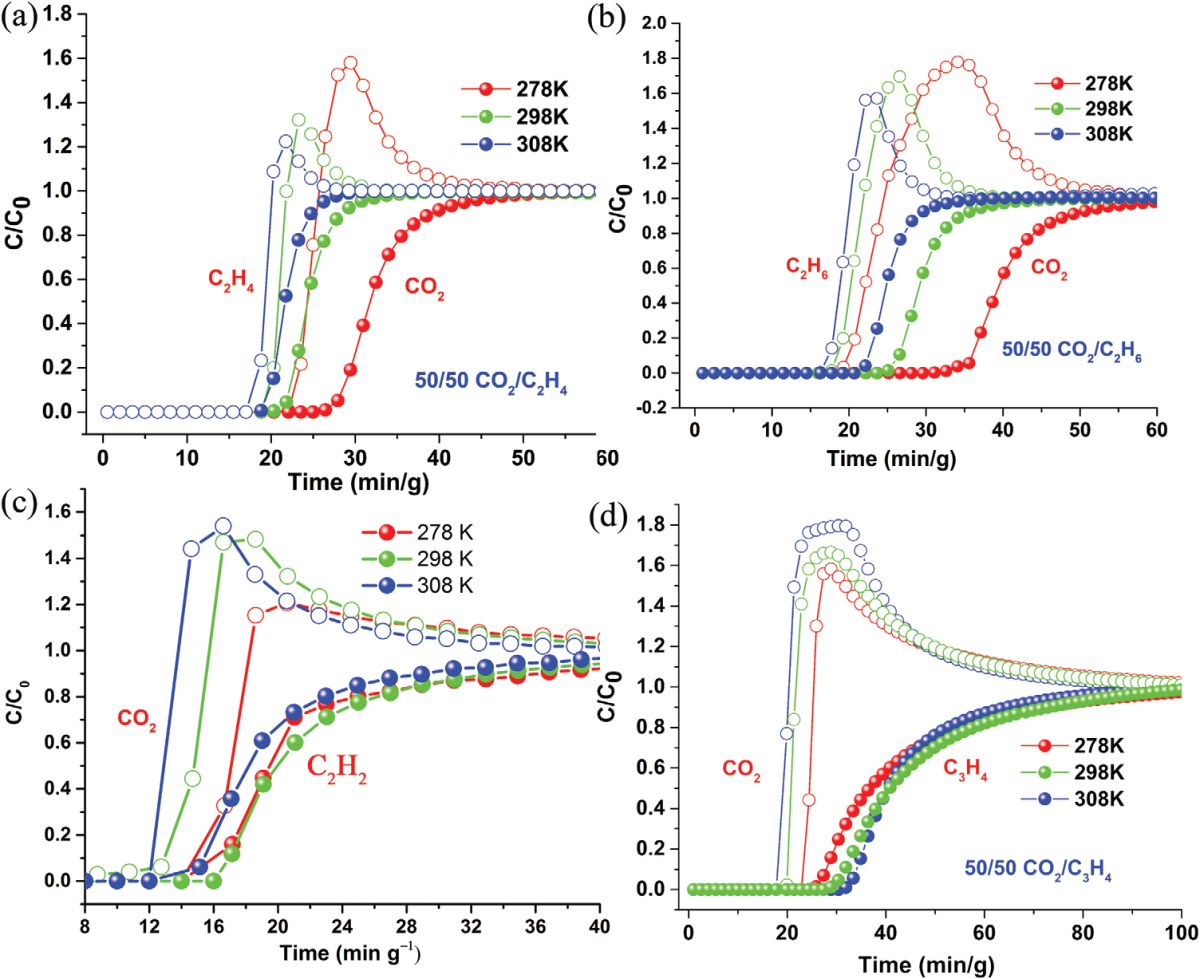
Experimental breakthrough curves of activated‐**1** for a) CO_2_/C_2_H_4_ (50/50, v/v), b) CO_2_/C_2_H_6_ (50/50, v/v), c) CO_2_/C_2_H_2_ (50/50, v/v), and d) CO_2_/C_3_H_4_ (50/50, v/v) at 278, 298, and 308 K, respectively.

The comparison of the CO_2_ isotherms with those of light hydrocarbons at diverse temperatures demonstrates possible temperature‐dependent separation (Figure S14, Supporting Information), experimental breakthrough curves are therefore measured. The CO_2_/C_2_H_4_, CO_2_/C_2_H_6_, CO_2_/C_2_H_2_, and CO_2_/C_3_H_4_ mixtures were selected, because it is important but challenging to separate these gas mixtures in view of their similar sizes and physical properties.^[^ ^52^ ^]^ For the CO_2_/C_2_H_4_ (50/50, v/v) and CO_2_/C_2_H_6_ (50/50, v/v) mixtures, the experimental breakthrough curves were measured using a column (180  × 3 mm) packed with activated‐**1** under a flow rate of 1.0 mL min^‒1^ at 278, 298, and 308 K. For both the mixtures, CO_2_ broke out after the hydrocarbons (**Figure** **5**a,b; Figures S15 and S16, Supporting Information). The dynamic capture amounts of CO_2_ decrease with increasing temperature while those of C_2_H_4_ and C_2_H_6_ decrease slightly. As a result, the best separation temperature for both the mixtures is 278 K among the three measured temperature. The the retention time lags change from ≈8 min g^‒1^ at 278 K to ≈2 min g^‒1^ at 308 K and ≈17 min g^‒1^ at 278 K to ≈6 min g^‒1^ at 308 for the CO_2_/C_2_H_4_ and CO_2_/C_2_H_6_ mixtures, respectively. For the CO_2_/C_2_H_2_ (50/50, v/v) and CO_2_/C_3_H_4_ (50/50, v/v) mixtures, the experimental breakthrough curves were measured under a flow rate of 2.0 mL min^‒1^. The results reveal that CO_2_ break out before the hydrocarbons for both the mixtures (Figure 5c,d; Figures S17 and S18, Supporting Information). The dynamic capture amount of C_2_H_2_ increases from 278 to 298 K and that of C_3_H_4_ increases with increasing temperature. As the dyanmic capture amounts of CO_2_ decrease with increasing temperature, the separation performances for these two mixtures at 298 and 308 K are higher than those at 278 K. Taking the CO_2_/C_2_H_2_ mixture for example, both CO_2_ and C_2_H_2_ broke out at ≈14 min g^‒1^ at 278 K. In contrast, CO_2_ shows a faster elution at ≈12 min g^‒1^ but C_2_H_2_ is retained until ≈16 min g^‒1^ at 298 K. At 308 K, CO_2_ and C_2_H_2_ broke out at ≈12 and 15 min g^‒1^, respectively. Therefore, the retention time lags change from ≈0 min g^‒1^ at 278 K to ≈3 min g^‒1^ at 298 and 308 K. The dynamic CO_2_ capture amounts at 298 and 308 K are ≈0.54 and ≈0.53 mmol g^‒1^, respectively, which are comparable to the equilibrium adsorption under similar conditions (0.59 mmol g^‒1^ at 298 K and 0.5 bar and 0.47 mmol g^‒1^ at 308 K and 0.5 bar). For the CO_2_/C_3_H_4_ mixture, the separation performance is improved by temperature more significantly. The retention time lags increase from ≈1 min g^‒1^ at 278 K to ≈14 min g^‒1^ at 308 K.

The dynamic CO_2_ capture amounts decreasing with increasing temperature is governed by thermodynamics, because the CO_2_‒framework affinity becomes weaker at higher temperatures. While the kinetically enhanced diffusions of light hydrocarbons at higher temperatures enhance the uptakes of the corresponding hydrocarbons, thus leading to unusual temperature‐dependent sorption selectivities as indicated by the calculated IAST (ideal adsorption solution theory) selectivities (Figure S19, Supporting Information) with the sorption isotherms. Consequently, unusual temperature‐dependent separations of CO_2_ from the corresponding hydrocarbons were achieved. The relative high‐temperature threshold may be attributed to the coordination of the pendant naphthimidazolylmethyl arms with Mn^2+^, which raises the energy required for vibration and swing of the arms.

**Figure 6:**
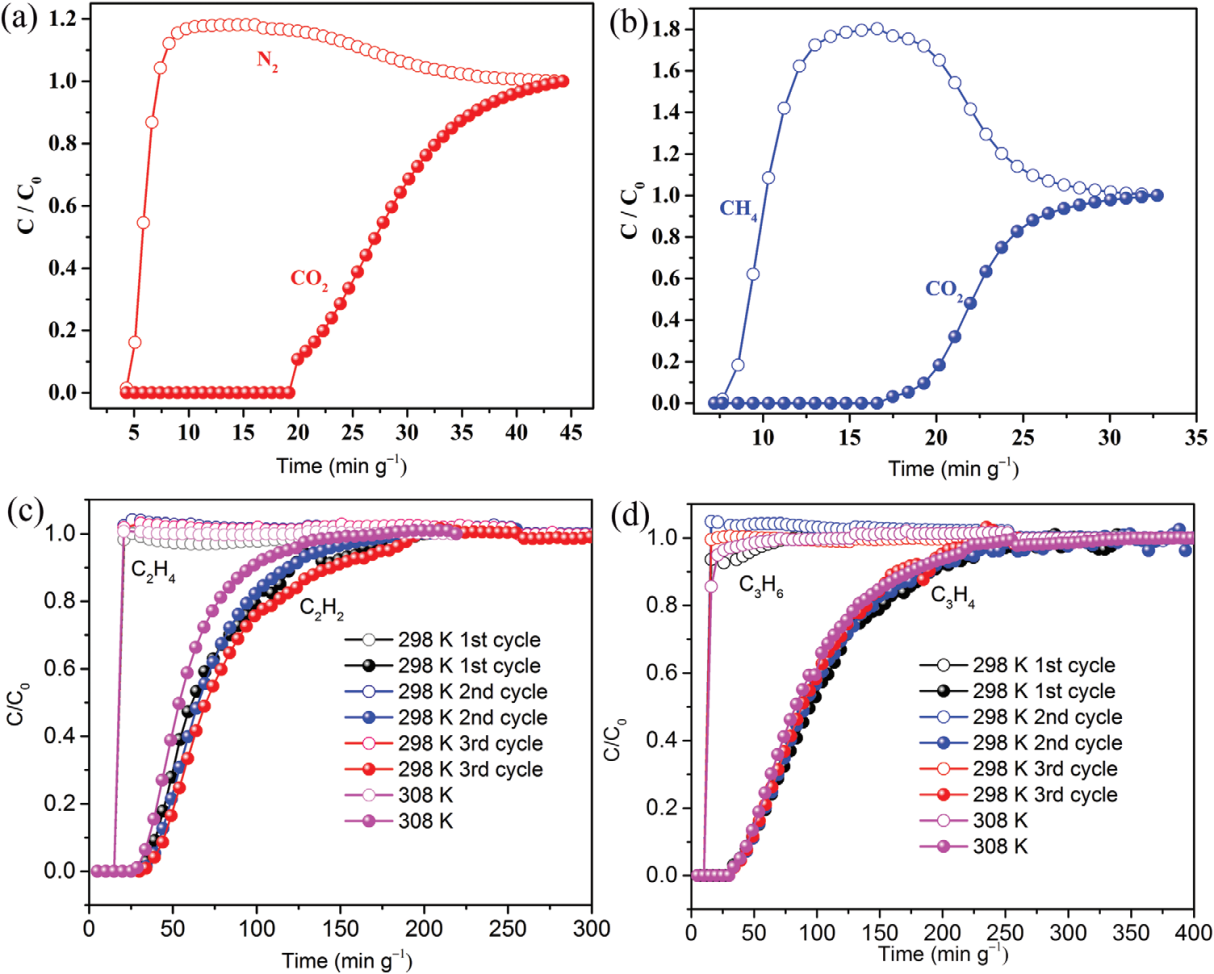
Experimental breakthrough curves of activated‐**1** for a) CO_2_/N_2_ (15/85, v/v), b) CO_2_/CH_4_ (50/50, v/v), c) C_2_H_2_/C_2_H_4_ (1/99, v/v), and d) C_3_H_4_/C_3_H_6_ (1/99, v/v).

The sorption isotherms also reveal that activated‐**1** selectively captured CO_2_ over CH_4_ and N_2_ (Figure S20a,b, Supporting Information), C_2_H_2_ over C_2_H_4_ (Figure S20c, Supporting Information), and C_3_H_4_ over C_3_H_6_ (Figure S20d, Supporting Information), therefore breakthrough curves for the binary mixtures of CO_2_/N_2_ (15/85, v/v), CO_2_/CH_4_ (50/50, v/v), C_2_H_2_/C_2_H_4_ (1/99, v/v), and C_3_H_4_/C_3_H_6_ (1/99, v/v) were also measured at 298 K. For the CO_2_/N_2_ and CO_2_/CH_4_ mixtures, N_2_ and CH_4_ pass through the packed column quickly, whereas CO_2_ does not break through until ≈19 min g^–1^ (with N_2_ **Figure** **6**a,) and ≈17 min g^–1^ (with CH_4_ Figure 6b). For the C_2_H_2_/C_2_H_4_ and C_3_H_4_/C_3_H_6_ mixtures, C_2_H_4_ and C_3_H_6_ pass through the packed column at ≈10 min g^‒1^ and ≈15 min g^‒1^, respectively, and the retention times for C_2_H_2_ and C_3_H_4_ are both ≈30 min g^‒1^ (Figure 6c,d). These results indicate **1** is potential for selective CO_2_ capture from industrial off‐gas and alkyne/olefin separation.

## 5 Conclusion

In summary, driven by hydrogen bonding and *π*–*π* stacking interaction a flexible self‐assembly arising from dimeric [Mn_2_L_2_Cl_2_]^2+^ bearing pendant naphthimidazolylmethyl arms has been assembled. The self‐assembly features 1D channels which are composed of octahedral cages with small gates. Unusual diffusion‐regulatory adsorption of light hydrocarbons was achieved for the dynamic pendant naphthimidazolylmethyl arms, thus leading to the temperature‐dependent separation of CO_2_ and the corresponding hydrocarbons. As illustrated here, the local dynamics of organic constituents can enable the diffusion control of guest molecules, which provides opportunities for a wide range of porous materials for efficient storage and separation.

## Conflict of Interest

The authors declare no conflict of interest.

## Supporting information

Supporting Information

Supporting Information

## Data Availability

The data that support the findings of this study are available from the corresponding author upon reasonable request.
