# Supplementary material for: Temperature‐Dependent Separation of CO2 from Light Hydrocarbons in a Porous Self‐Assembly of Vertexes Sharing Octahedra
Source: Adv Sci (Weinh). 2024 Feb 2;11(14):2308028. doi: 10.1002/advs.202308028 (PMC11005747; doi:10.1002/advs.202308028)
Supplement: Supplementary file 1 — Supporting Information [file ADVS-11-2308028-s002.pdf]

ADVANCED SCIENCE
Open Access

Supporting Information

for *Adv. Sci.*, DOI 10.1002/advs.202308028

Temperature-Dependent Separation of CO_2 from Light Hydrocarbons in a Porous Self-Assembly of Vertexes Sharing Octahedra

Shun Li, Qing Li, Ting Chen, Zhen-Yu Ji, Guo-Ling Li, Ming-Yan Wu, Li-Yi Meng, Zi-Ang Nan, Wei Wang, Zhu Zhuo, Fengru Fan and You-Gui Huang**

Supporting Information

Temperature-Dependent Separation of CO_2 from Light Hydrocarbons in a Diffusion-Regulatory Porous Self-Assembly

Shun Li,[a],[b]† Qing Li,[a],[b]† Ting Chen,[a],[b]† Zhen-Yu Ji,[c] Guo-Ling Li,[a],[b] Ming-Yan Wu,[c] Li-Yi, Meng,[a],[b] Zi-Ang Nan,[a],[b] Wei Wang,[a],[b] Zhu Zhuo,[a],[b] Fengru Fan,[d] and You-Gui Huang[a],[b]**

Correspondence to: wumy@fjirsm.ac.cn, yghuang@fjirsm.ac.cn

[a] CAS Key Laboratory of Design and Assembly of Functional Nanostructures, and Fujian Provincial Key Laboratory of Nanomaterials, Fujian Institute of Research on the Structure of Matter, Chinese Academy of Sciences, Fuzhou, Fujian, 350002, China

[b] Xiamen Key Laboratory of Rare Earth Photoelectric Functional Materials, Xiamen Institute of Rare Earth Materials, Haixi Institutes, Chinese Academy of Sciences, Xiamen, Fujian, 361021, China

[c] State Key Laboratory of Structure Chemistry, Fujian Institute of Research on the Structure of Matter, Chinese Academy of Sciences, Fuzhou, Fujian, 350002, China

[d] State Key Laboratory of Physical Chemistry of Solid Surfaces, Xiamen University, Xiamen, Fujian, 361005, China

Table of Contents:

S1. Materials and methods
- **S1.1** Starting materials
- **S1.2** Physical measurements
- **S1.3** Crystallography
- **S1.4** Breakthrough measurements
- **S1.5** Calculation of Q_{st} by molecular dynamics simulation
- **S1.6** Calculation of the selectivities by IAST.
- **S1.7** Evalution of the diffusion rates of C_2H_2 in activated-**1**

S2. Syntheses
S3. Structure of **1**
S4. Thermal and chemical stabilities and regeneration
S5. Gas adsorption properties of activated-**1**.
S6. References

S1. Materials and Methods

S1.1 Starting materials

The ligand L (tris(2-naphthimidazolylmethyl)amine) was prepared according to the procedure reported in literature.[S1] All other reagents were purchased from commercial sources and used without purification.

S1.2 Physical measurements

Thermogravimetric analyses (TGA) were performed using a TG/DTA6300 system at a rate of 10 °C/min. Powder X-ray diffraction (PXRD) patterns were obtained on a Rigaku 2100 diffractometer using Cu-*K*α radiation with flat plate geometry. High-resolution Electrospray Ionization Time of Flight Mass Spectrometry (ESI-TOF-MS) measurements were performed on a DECAX-30000 LCQ Deca XP system. Scanning electron microscopy (SEM) was performed on a Hitachi SU1510 scanning electron microscope. The sample of **1** was desolvated at 100 °C under high vacuum for 5 h to remove the guest molecules before gas sorption measurements. Gas sorption isotherms of activated-**1** were measured on a Micromeritics ASAP 2020 surface area analyzer or on a 3Flex instrument. Both the instruments are produced by Micromeritics Instrument Corporation for conventional gases sorption measurements. The maximum vacuum degree of 3Flex is as low as 1.3×10^{-9} bar, while thar for ASAP 2020 is only about 1.0×10^{-5} bar. In addition, the equilibrium time for isotherm measurement of 3Flex is automatically set to longer than that of ASAP 2020.

S1.3 Crystallography

Single-crystal X-ray data were harvested on a Bruker D8 Venture diffractometer with Mo-K_α radiation at 200 K. Structures were solved using a direct method and refined by the full-matrix least-squares technique on F^2 with SHELXTL 2014 program.[S2] The hydrogen atoms are geometrically generated and refined using a riding model. The PLATON/SQUEEZE procedures[S3] were used to treat the highly disordered solvents in the voids of the structure of **1**. The X-ray crystallographic coordinates for structures reported in this article have been deposited at the Cambridge Crystallographic Data Centre (CCDC), under deposition numbers CCDC 2300877−2300882. These data can be obtained free of charge from The Cambridge Crystallographic Data Centre via www.ccdc.cam.ac.uk/data_request/cif. Detail crystallographic data are listed in Table S1.

S 1.4 Breakthrough measurements.

Breakthrough experiments were performed on a fixed bed. A stainless-steel column with a length of 180 mm and an internal diameter of 3 mm was used for sample packing. The flow rates of all gases are regulated by mass flow controllers, and the effluent gas stream from the column is monitored by gas chromatography (GC) detector. All measurements were performed following a protocol established by literatures. The crystals of **1** (0.896 g) were packed into a stainless-steel column tightly. After the column was activated with the He flow under 100 °C for 5 h, a mixture flow was dosed into the column. Breakpoints were determined when the first peak was detected.

S1.5 Calculation of the isosteric heat of adsorption (Q_{st}).

$$\ln P = \ln N + \sum_{i=0}^{m} a_i N^i + \sum_{i=0}^{n} \binom{n}{k} b_i N^i$$

$$Q_{ST} = -R \sum_{i=0}^{m} a_i N^i$$

A virial-type expression of the above form was used to fit the combined isotherm data of activated-**1** at 273, 298, and 308 K, where P is the pressure described in mmHg, N is the adsorbed amount in mg/g, T is the temperature in K, a_i and b_i are virial coefficients, and m and n are the number of coefficients used to describe the isotherms. Q_{st} is the coverage-dependent heat of adsorption and R is the universal gas constant.

S1.6 Calculation of the selectivities by IAST.

Single-component gas equilibrium adsorption isotherms were fitted with the Langmuir–Freundlich model, given by the following equation:

$$N = A_1 \frac{b_1 p^{c_1}}{1 + b_1 p^{c_1}}$$

where N is the amount of adsorbed gas (mmol g^{-1}), p is the bulk gas phase pressure (atm), A_1 is the adsorption saturation capacities for site 1 (mmol g^{-1}), b_1 is the affinity coefficient of site 1 (1/kPa), c_1 is the Langmuir–Freundlich exponent (dimensionless) for the adsorption sites A.

The parameters of A_1, b_1, and c_1 were used to predict the adsorption selectivities based on IAST, which is finally defined as:

$$S_{\frac{1}{2}} = \left(\frac{x_1}{x_2}\right)\left(\frac{y_2}{y_1}\right)$$

where S is the ideal selectivity of component 1 over component 2, x_i and y_i are the mole fractions of component i (i = 1, 2) in the adsorbed and bulk phases, respectively.

S1.7 Evalution of the diffusion rates of C_2H_2 in activated-1

The CO_2-adsorbed and C_2H_2-adsorbed structures of activated-**1** were simulated by Grand Canonical Monte Carlo (GCMC), and the Molecular dynamics (MD) method has been used to analyze the mean square displacement (MSD). The energy minimization and geometry optimization process of these models have been performed in the forcite module. And then, the annealing process of these molecular models has been performed to obtain stable conformations. Finally, MD simulations of these molecular models have been performed with a 10 ps dynamic simulation under equilibrium run with constant volume and temperature (NVT) and a 5 ps dynamic simulation under the constant volume and energy (NVE) ensemble. After MD simulation, the diffusion coefficient have been analyzed in the forcite analysis module. All the above simulation results have been completed by Materials Studio software.

Table S1. Crystallographic data of **1** and activated-**1**.

Compounds	**1**	activated-**1** (100 K)	activated-**1** (195 K)
Formula	$C_{90}H_{60}Cl_4Mn_2N_{20}$	$C_{90}H_{60}Cl_4Mn_2N_{20}$	$C_{90}H_{60}Cl_4Mn_2N_{20}$
Formula weight	1673.26	1673.26	1673.26
Temp. (*K*)	200	100	195
Crystal System	Trigonal	Trigonal	Trigonal
Space group	$R\bar{3}$	$R\bar{3}$	$R\bar{3}$
a (Å)	28.042(3)	27.8273(8)	27.8962(6)
c (Å)	61.129(14)	60.594(3)	61.033(2)
V(Å^3)	41628(13)	40635(3)	41133(2)
Z	18	18	18
$\rho_{cal.}$(g cm^{-3})	1.201	1.231	1.216
μ	0.442	0.453	0.447
F(000)	15444	15444	15444
θ *range* (°)	1.865–27.491	1.881–27.512	2.002–27.513
Reflections ($I > 2\sigma$)	13528	10550	8965

$R_1(I > 2\sigma)$	0.1203	0.1486	0.1185
$wR_2(all)$	0.3319	0.3959	0.3323
GOF on F^2	1.063	1.115	1.045
CCDC#	2300877	2300878	2300879

Compounds	activated-**1** (270 K)	activated-**1** (298 K)	activated-**1** (308 K)
Formula	$C_{90}H_{60}Cl_4Mn_2N_{20}$	$C_{90}H_{60}Cl_4Mn_2N_{20}$	$C_{90}H_{60}Cl_4Mn_2N_{20}$
Formula weight	1673.26	1673.26	1673.26
Temp. (*K*)	270	298	308
Crystal System	Trigonal	Trigonal	Trigonal
Space group	$R\bar{3}$	$R\bar{3}$	$R\bar{3}$
a (Å)	27.9348(3)	27.9479(6)	27.9569(3)
c (Å)	61.4151(18)	61.5838(11)	61.6602(19)
V(Å^3)	41504.6(15)	41657.7(19)	41736.2(16)
Z	18	18	18
$\rho_{cal.}$(g cm^{-3})	1.205	1.201	1.198
μ	0.443	0.441	0.441
F(000)	15444	15444	15444
θ *range* (°)	1.990–27.579	1.984–27.502	2.250–27.514
Reflections $(I > 2\sigma)$	9206	8281	7979
$R_1(I > 2\sigma)$	0.0742	0.0963	0.0805
$wR_2(all)$	0.2411	0.3206	0.2771
GOF on F^2	1.039	1.016	1.020
CCDC#	2300880	2300881	2300882

[a] $R = \sum||F_0|-|F_c||)/\sum|F_0|$

[b] $wR = |\sum w(F_0-F_c)^2/\sum w(F_0{}^2)^2|^{1/2}$

S2. Syntheses

Synthesis of $\{[(Mn_2L_2Cl_2)Cl_2]\cdot 2(btcn)\}_n$ (**1**):

$MnCl_2\cdot 4H_2O$ (135 mg, 0.68 mmol), tris(2-naphthimidazole methyl) amine (L) (27 mg, 0.05 mmol), and *1,3,5*-benzenetricarbonitrile (btcn) (13 mg, 0.05 mmol) were dissolved in a MeOH/acetone mixture (12 mL, 2:10 v/v) giving rise to a yellow solution. The filtrate was left undisturbed at room temperature for several days. Pale yellow prism-shaped crystals of **1** were harvested. Yield: 65% based on L.

S3. Structures of 1.

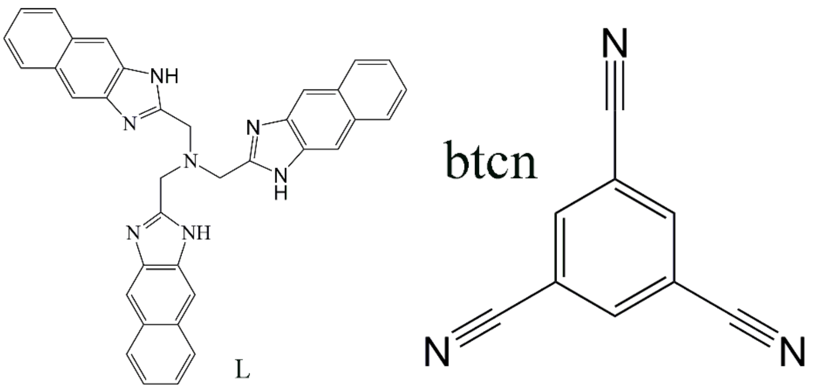

Figure S1. Molecular structures of L and btcn.

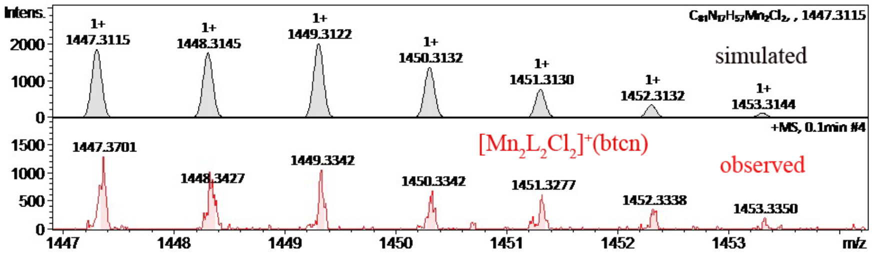

Figure S2. High resolution mass spectrum (HR-MS) of $\{[Mn_2L_2Cl_2](btcn)\}^+$ obtained from the crystallizing solution of **1**.

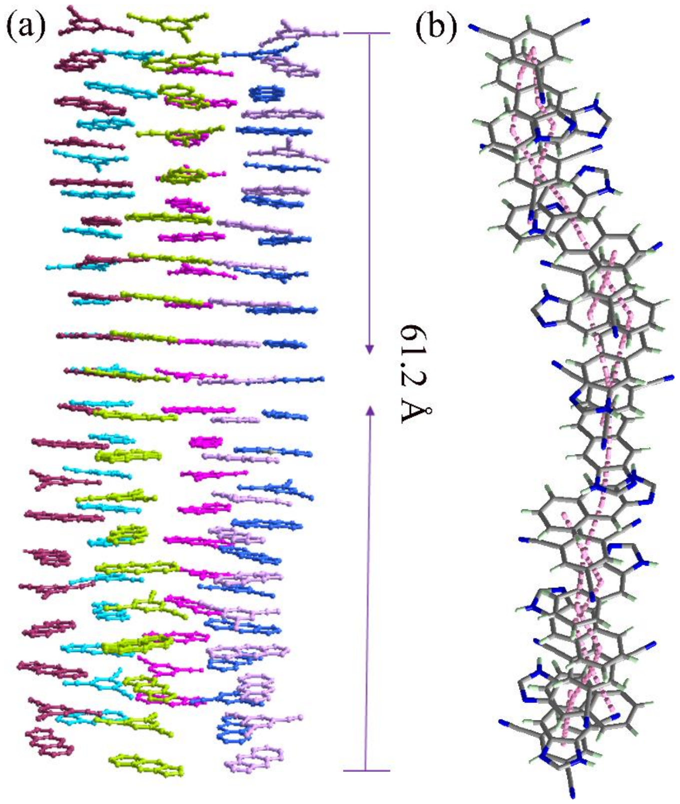

Figure S3. (a) Side view of the channel wall formed by hexagonally aligned π-stacked helical columns, the different helical columns are shown in different colors. (b) One of the π-stacked helical columns possessing a pitch of 61.2 Å.

S4. Thermal and chemical stabilities and regeneration.

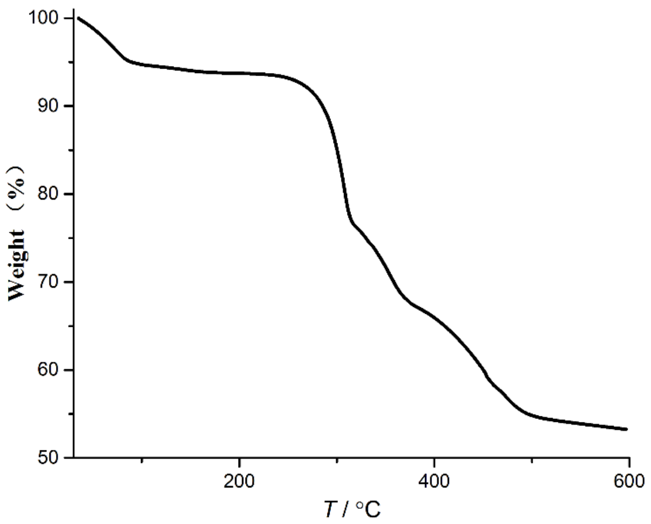

Figure S4. TGA for **1**. TG analyses show a weight loss below 100 °C which corresponds to the loss solvent molecules.

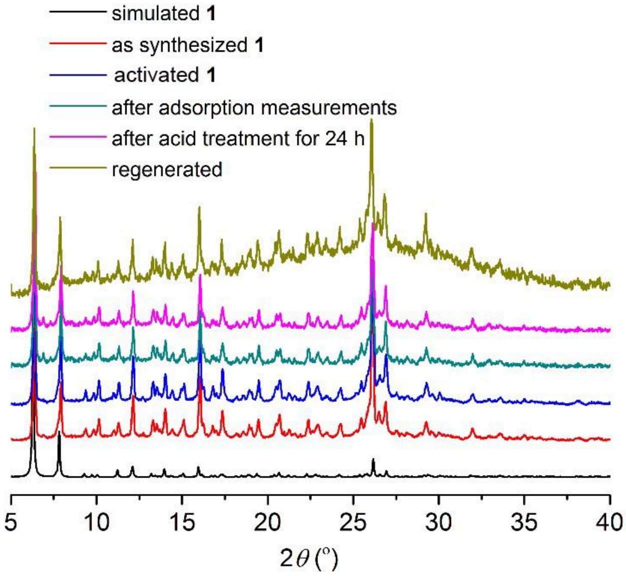

Figure S5. PXRD patterns of **1** showing the exceptional thermal and chemical stabilities and the exceptional regeneration property. The desolvated sample was activated at 100 °C under vacuum for 10 h.

Regeneration of 1:

1 (40.0 mg) was dissolved in a MeOH/acetone mixture (12 mL, 2/10 v/v/) at room temperature. The resulting yellow solution was left undisturbed at room temperature for several days. Yellow prism-shaped crystals of **1** were recovered.

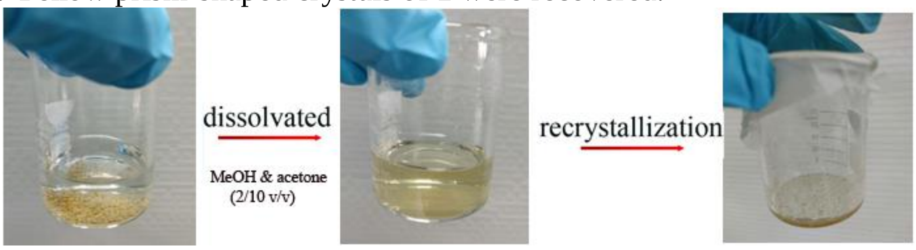

Figure S6. Scheme showing the regenerating process of **1**.

S5. Gas adsorption properties of activated-**1**.

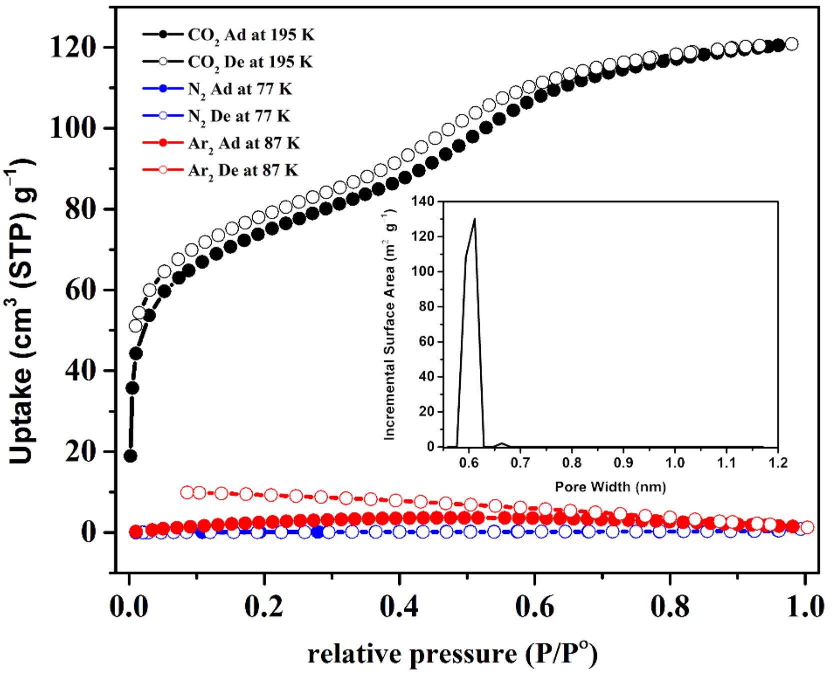

Figure S7. The N_2 sorption data at 77 K, Ar_2 sorption data at 87 K, and CO_2 sorption data at 195 K of desolvated-**1**, inset): pore size distribution of activated-**1**. The pore volume was calculated based on the CO_2 uptake at $P/P° = 0.96$, and the pore size distribution was extracted with the Non-Local Density Functional Theory (NLDFT) method[S4].

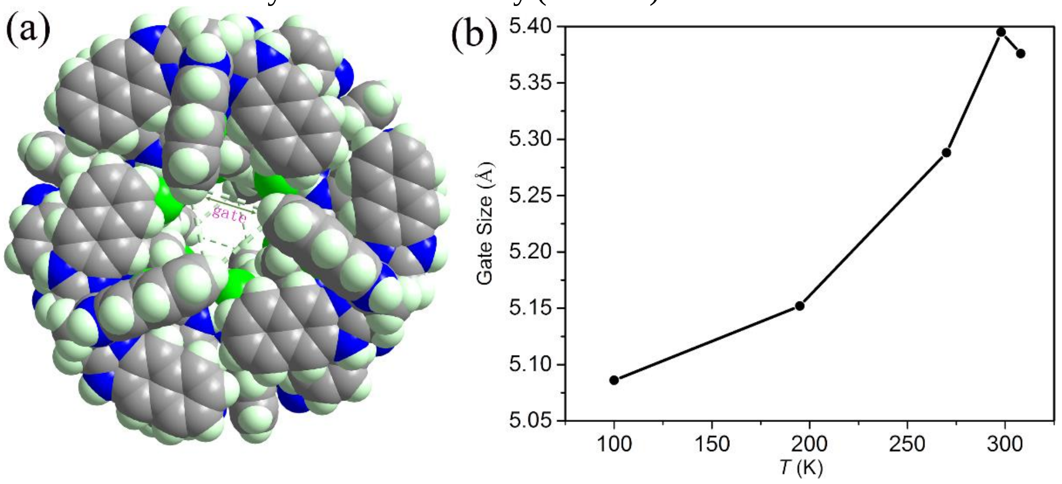

Figure S8. (a) The triangular gate of the channels in **1**. (b) The gate size increasing with temperature. (The size is described using the shortest H···H distance betweem adjacent naphthimidazolylmethyl arms).

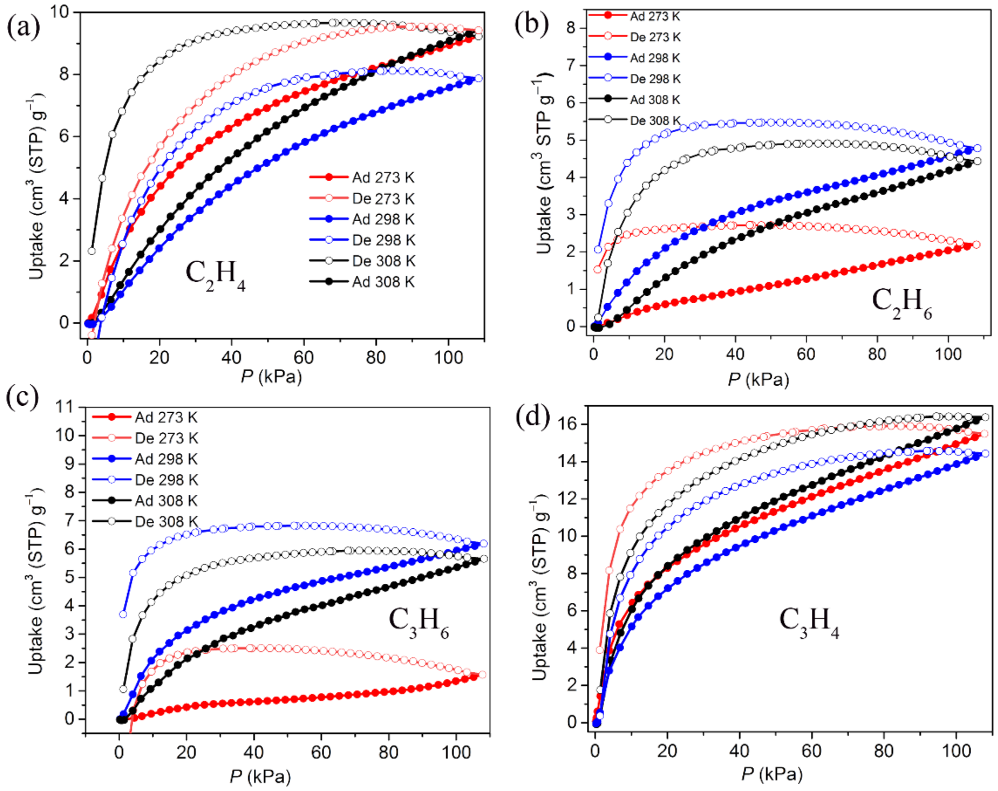

Figure S9. The sorption isotherms of ethylene (a), ethane (b), propylene (c), and propyne (d) measured on ASAP 2020.

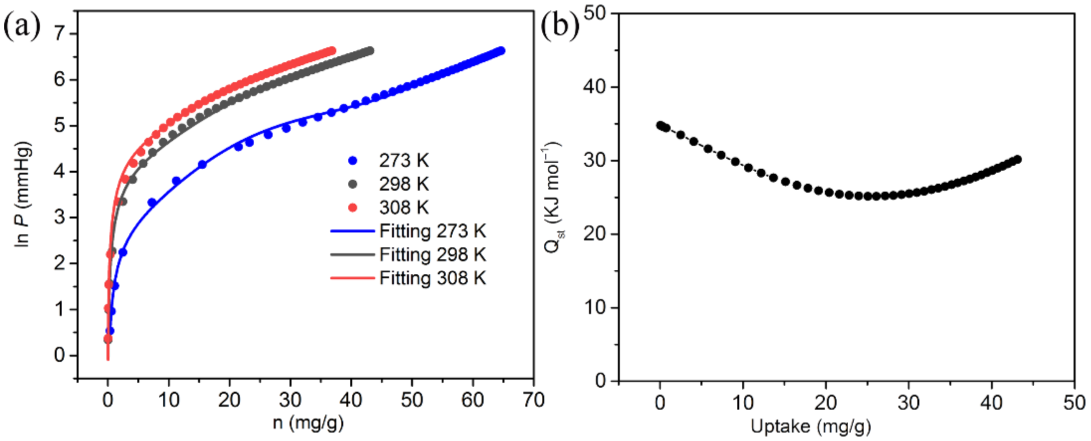

Figure S10. (a) The virial fitting of the adsorption of activated-**1** towards CO_2. (d) The isosteric heats of adsorption (Q_{st}) for CO_2 on activated-**1** calculated by the virial method.

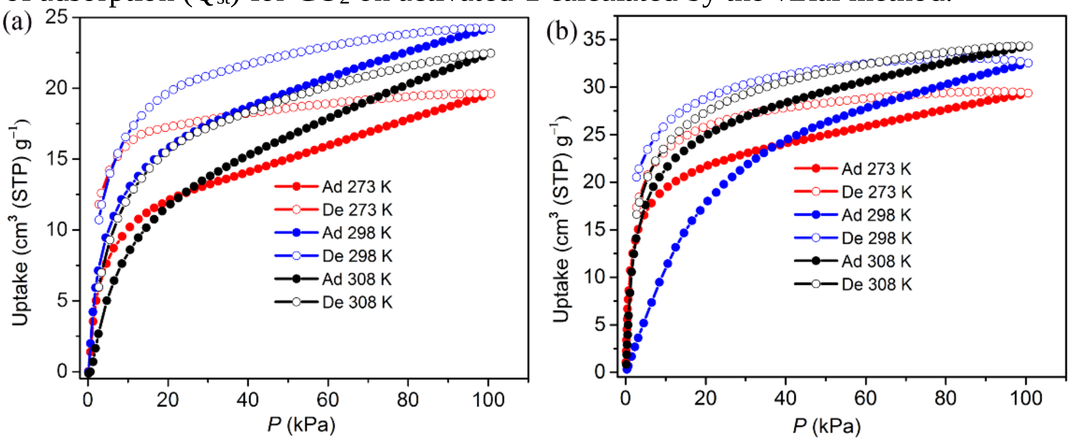

Figure S11. The sorption isotherms of propylene (a) and propyne (b) measured on 3Flex.

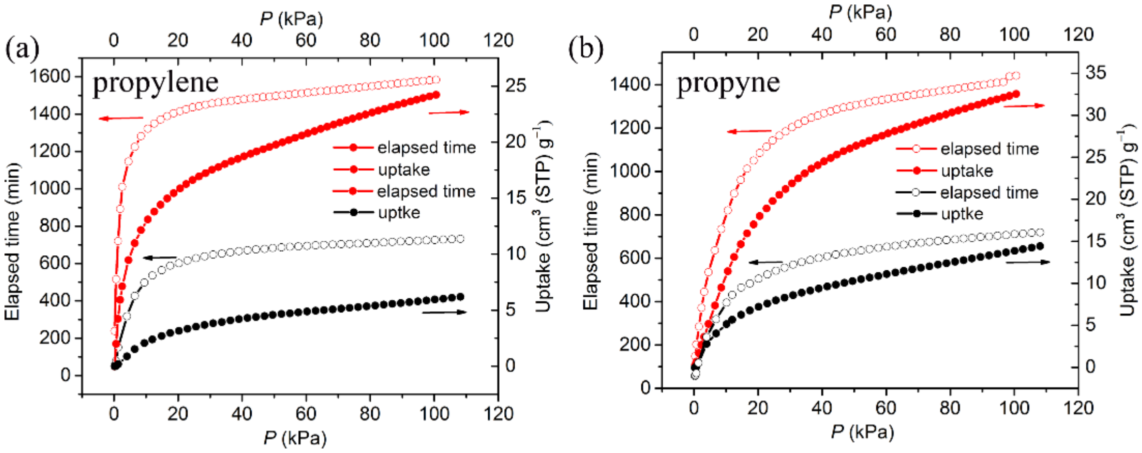

Figure S12. The propylene (a) and propyne (b) adsorption isotherms accompanying with elapsed times at 298 K.

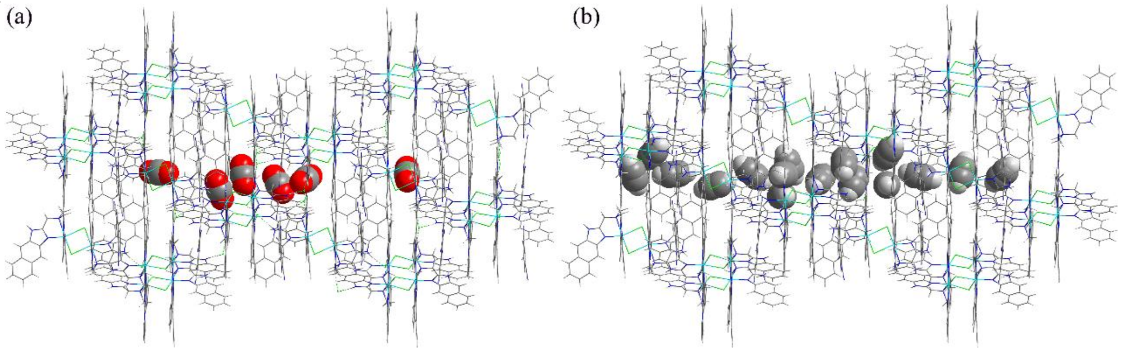

Figure S13. The calculated CO_2-adsorbed (a) and C_2H_2-adsorbed (b) structures of **1**.

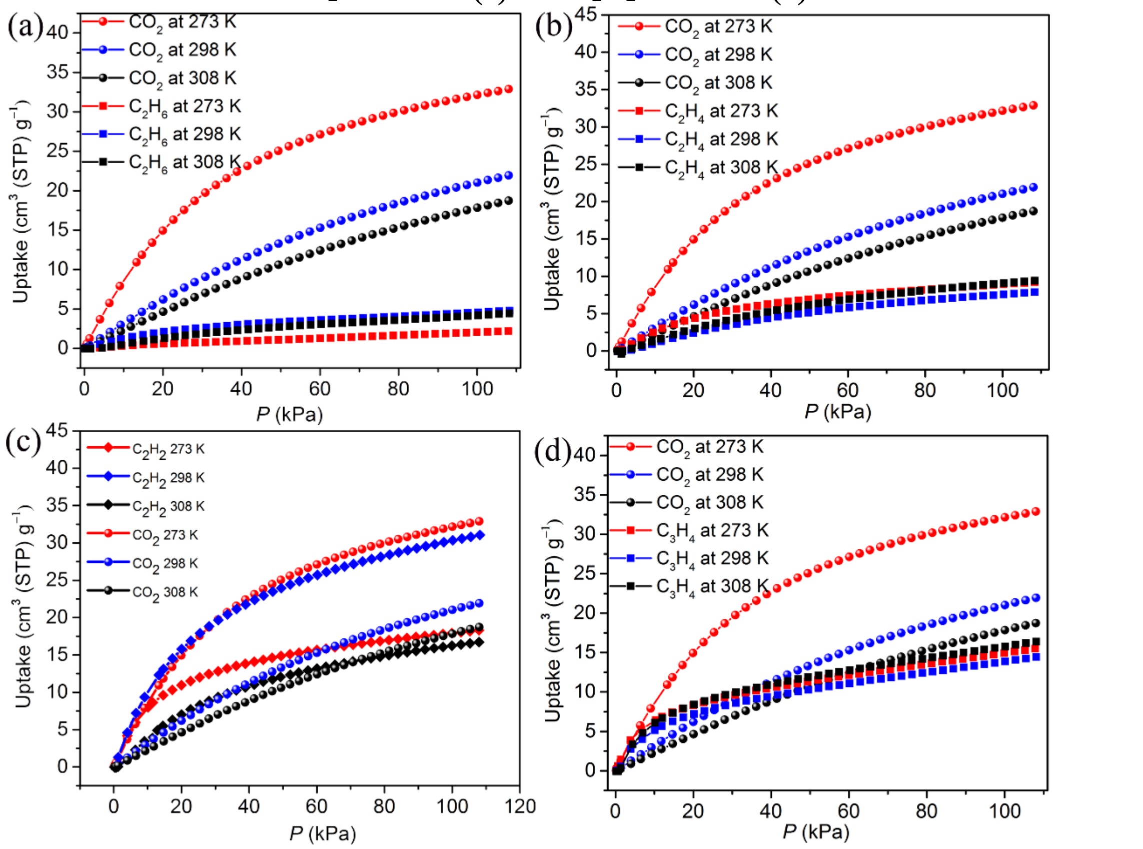

Figure S14. Comparison of the sorption isotherms of CO_2 with ethane (a), ethylene (b), acetylene (c), and propyne (d) at diverse temperatures.

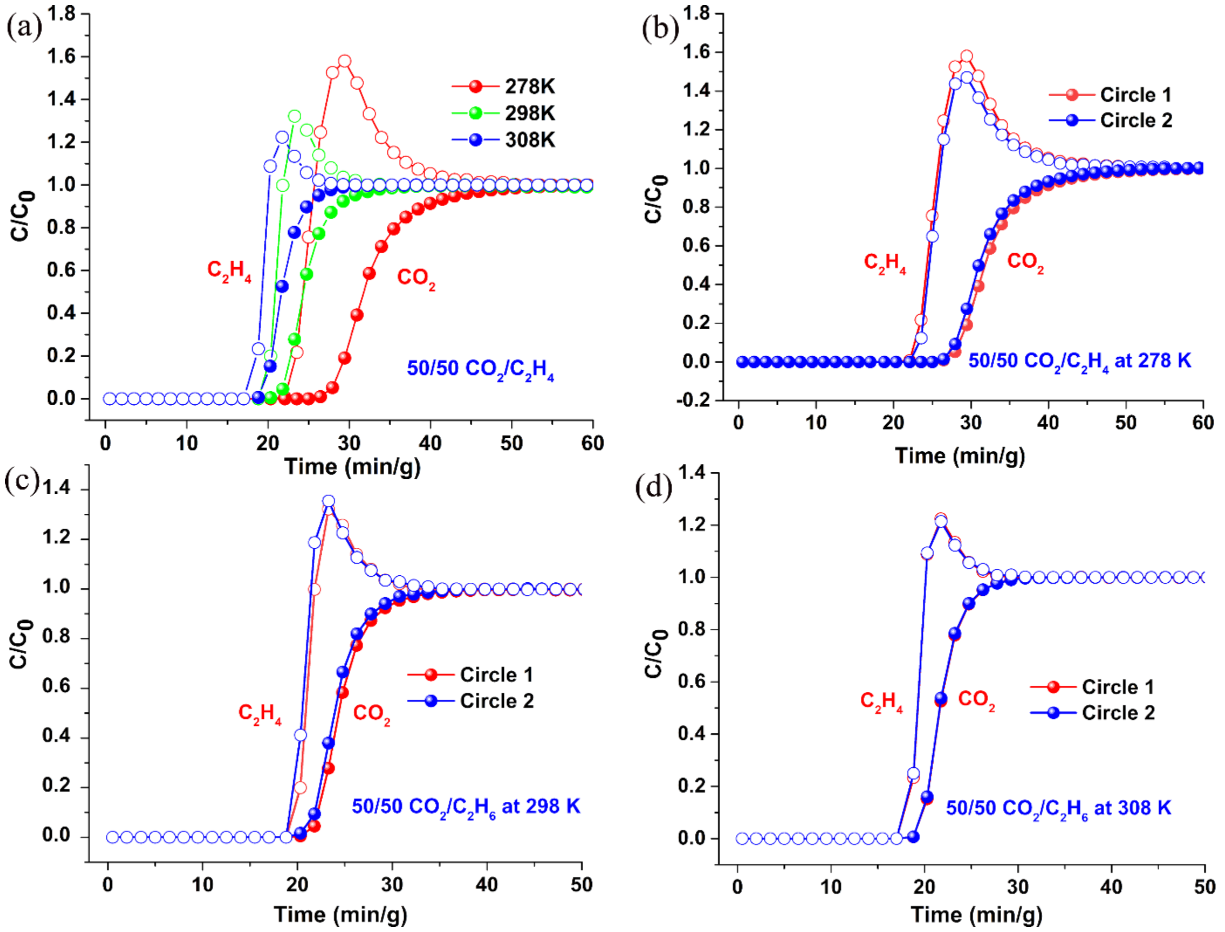

Figure S15. Experimental breakthrough curves of activated-**1** for CO_2/C_2H_4 (50/50, v/v) at 278, 298, and 308 K.

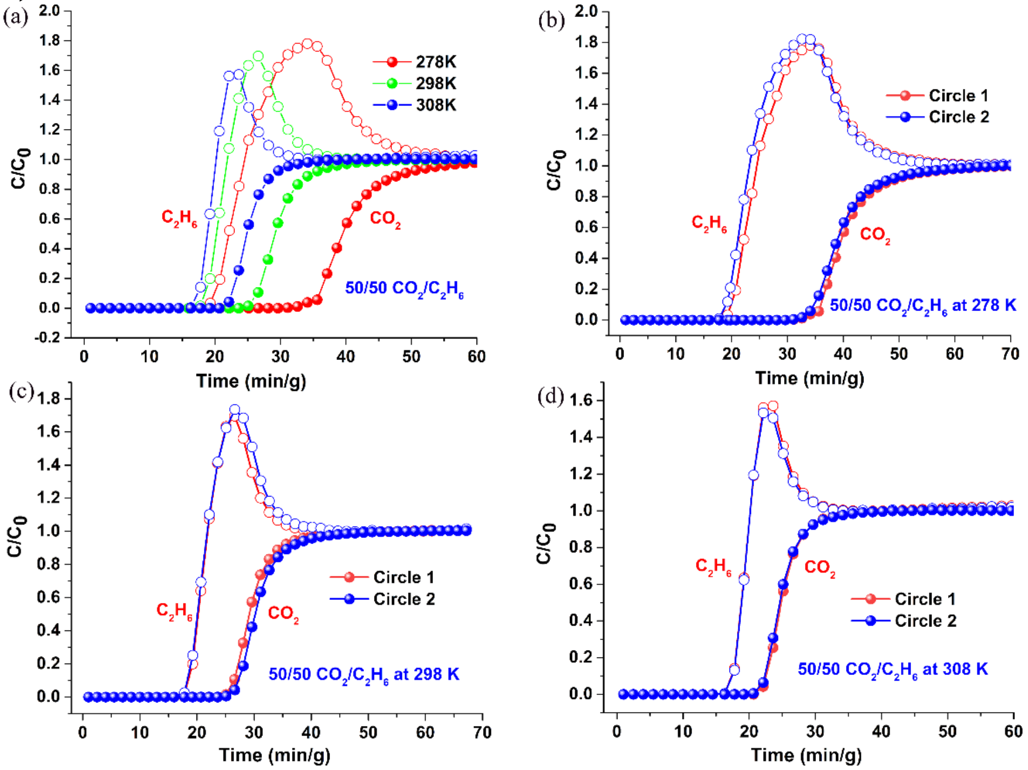

Figure S16. Experimental breakthrough curves of activated-**1** for CO_2/C_2H_6 (50/50, v/v) at 278, 298, and 308 K.

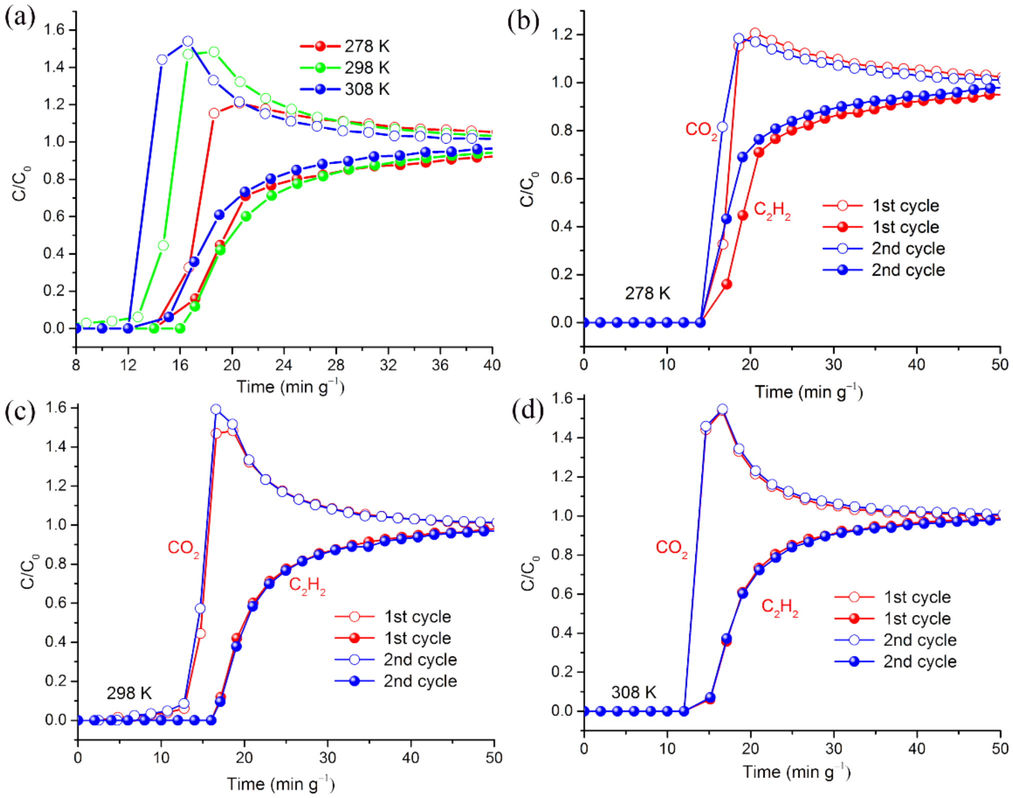

Figure S17. Experimental breakthrough curves of activated-**1** for CO_2/C_2H_2 (50/50, v/v) at 278, 298, and 308 K.

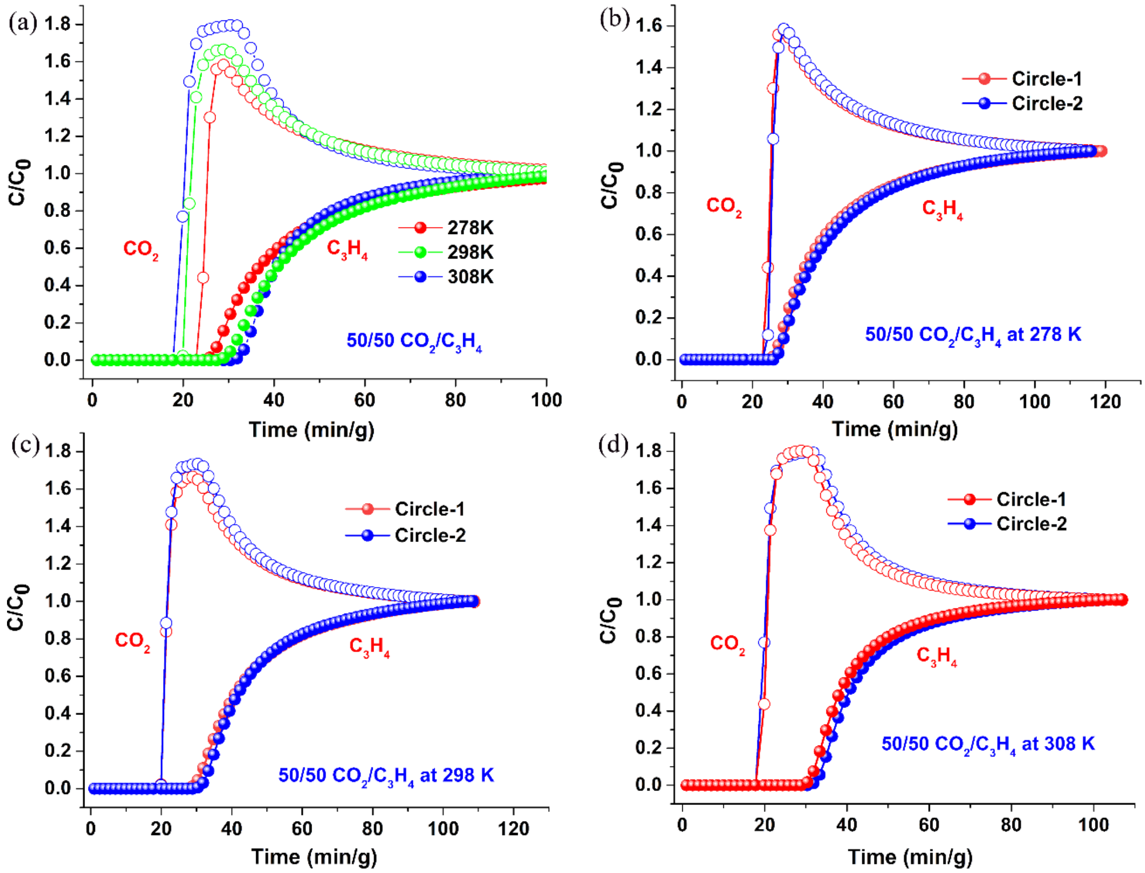

Figure S18. Experimental breakthrough curves of activated-**1** for CO_2/C_3H_4 (50/50, v/v) at 278, 298, and 308 K.

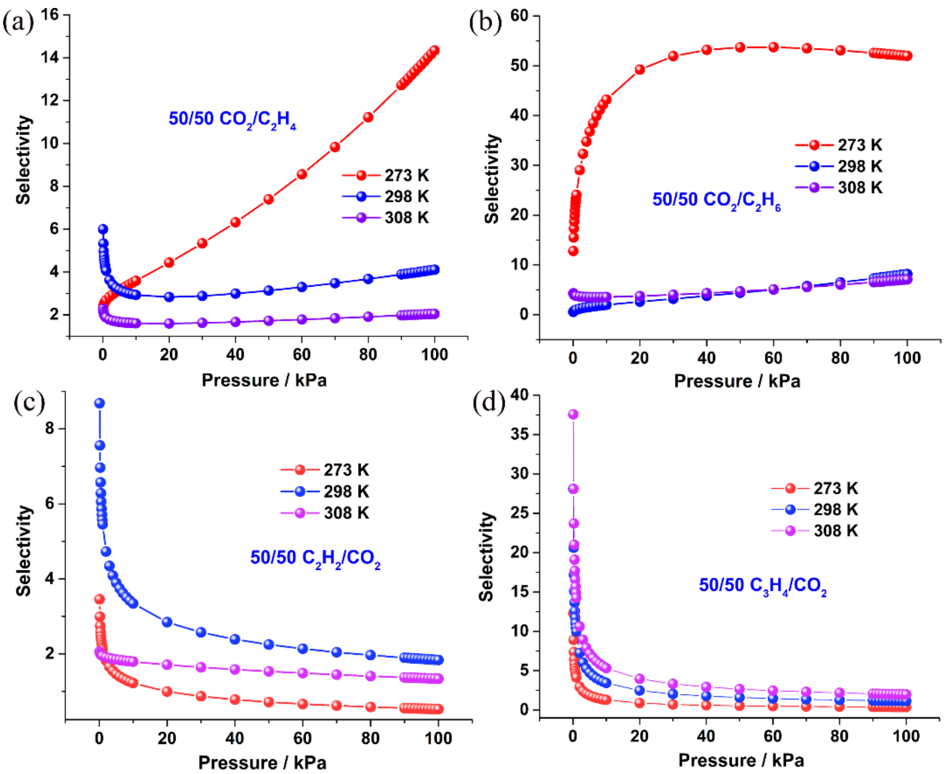

Figure S19. The calculated IAST selectivities for CO_2/C_2H_4 (a), CO_2/C_2H_6 (b), CO_2/C_2H_2 (c), and CO_2/C_3H_4 (d) at different temperatures.

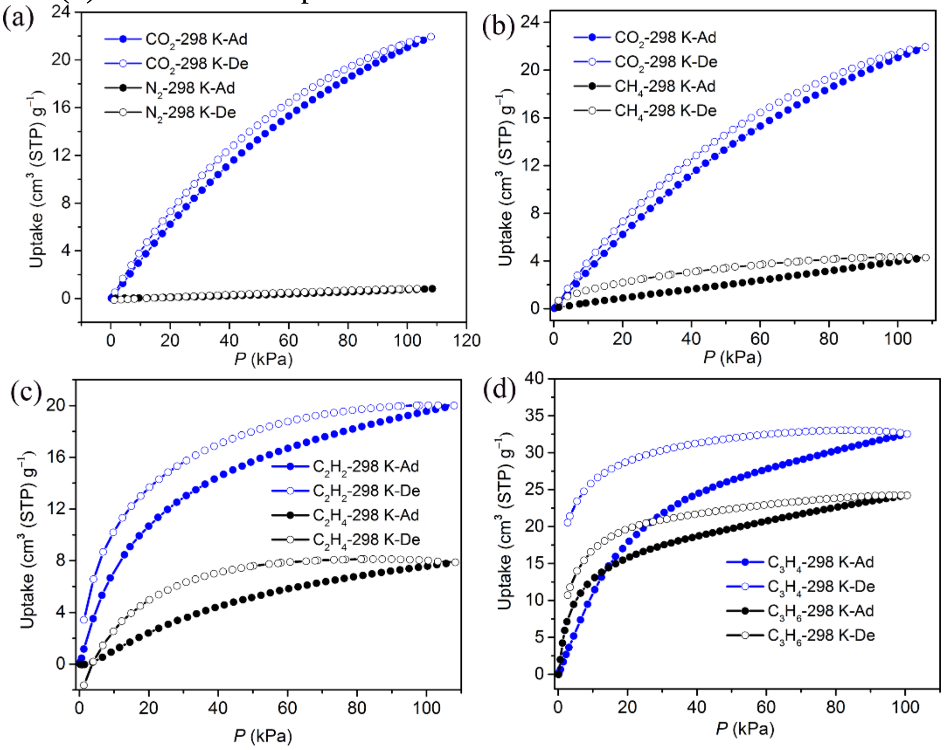

Figure S20. Comparison of the sorption isotherms of CO_2 with N_2 (a) and methane (b) at 298 K. (c) Comparison of the sorption isotherms of ethylene and acetylene at 298 K. (d) Comparison of the sorption isotherms of propylene and propyne at 298 K measured on 3Flex.

S6. References

S1. V. O. Rodionov, S. I. Presolski, S. Gardinier, Y. H. Lim, M. G. Finn, *J. Am. Chem. Soc.* **2007**, *129*, 12696.
S2. G. M. Sheldrick, *Acta Cryst. C*. **2015**, *71*, 3.
S3. A. L. Spek, *J. App. Cryst.* **2003**, *36*, 7.
S4. F. S. Tang, R. B. Lin, R. G. Lin, J. C. G. Zhao, B. L. Chen, *J. Solid State Chem.* **2018**, *258*, 346.
