# Supplementary material for: Temperature‐Dependent Separation of CO2 from Light Hydrocarbons in a Porous Self‐Assembly of Vertexes Sharing Octahedra
Source: Adv Sci (Weinh). 2024 Feb 2;11(14):2308028. doi: 10.1002/advs.202308028 (PMC11005747; doi:10.1002/advs.202308028)

checkCIF/PLATON report

You have not supplied any structure factors. As a result the full set of tests cannot be run.

THIS REPORT IS FOR GUIDANCE ONLY. IF USED AS PART OF A REVIEW PROCEDURE FOR PUBLICATION, IT SHOULD NOT REPLACE THE EXPERTISE OF AN EXPERIENCED CRYSTALLOGRAPHIC REFEREE.

No syntax errors found. CIF dictionary Interpreting this report

Datablock: 5

Bond precision: C-C = 0.0103 A Wavelength=0.71073

Cell:	a=27.9348(3)	b=27.9348(3)	c=61.4151(18)
	alpha=90	beta=90	gamma=120
Temperature:	270 K		

	Calculated	Reported
Volume	41504.7(15)	41504.6(15)
Space group	R -3	R -3 :H
Hall group	-R 3	-R 3
Moiety formula	C36 H27 Cl Mn N7, C9 H3 N3, Cl	C90 H60 Cl4 Mn2 N20
Sum formula	C45 H30 Cl2 Mn N10	C90 H60 Cl4 Mn2 N20
Mr	836.63	1673.26
Dx,g cm-3	1.205	1.205
Z	36	18
Mu (mm-1)	0.443	0.443
F000	15444.0	15444.0
F000'	15470.87	
h,k,lmax	36,36,79	36,36,79
Nref	21380	21184
Tmin,Tmax	0.938,0.965	
Tmin'	0.936	

Correction method= Not given

Data completeness= 0.991 Theta(max)= 27.579

R(reflections)= 0.0742(9206) wR2(reflections)= 0.2411(21184)

S = 1.039 Npar= 1045

The following ALERTS were generated. Each ALERT has the format
test-name_ALERT_alert-type_alert-level.
Click on the hyperlinks for more details of the test.

Alert level A

```
PLAT602_ALERT_2_A Solvent Accessible VOID(S) in Structure ........          ! Check
```

Alert level B

```
PLAT112_ALERT_2_B ADDSYM Detects New (Pseudo) Symm. Elem         n        100 %Fit
PLAT112_ALERT_2_B ADDSYM Detects New (Pseudo) Symm. Elem         n        100 %Fit
PLAT112_ALERT_2_B ADDSYM Detects New (Pseudo) Symm. Elem         n        100 %Fit
PLAT113_ALERT_2_B ADDSYM Suggests Possible Pseudo/New  Space Group       R-3c Check
              Check Model Parameter Symmmetry for Reflection Data Support
```

Alert level C

```
RINTA01_ALERT_3_C  The value of Rint is greater than 0.12
            Rint given   0.124
PLAT020_ALERT_3_C The Value of Rint is Greater Than 0.12 .........      0.124 Report
PLAT026_ALERT_3_C Ratio Observed / Unique Reflections (too) Low ..        43% Check
PLAT042_ALERT_1_C Calc. and Reported MoietyFormula Strings  Differ     Please Check
PLAT052_ALERT_1_C Info on Absorption Correction Method   Not Given     Please Do !
PLAT234_ALERT_4_C Large Hirshfeld Difference C86      --C99      .       0.16 Ang.
PLAT234_ALERT_4_C Large Hirshfeld Difference C100     --C102     .       0.20 Ang.
PLAT234_ALERT_4_C Large Hirshfeld Difference C85      --C96      .       0.17 Ang.
PLAT241_ALERT_2_C High   'MainMol' Ueq as Compared to Neighbors of        C49 Check
PLAT241_ALERT_2_C High   'MainMol' Ueq as Compared to Neighbors of       C109 Check
PLAT241_ALERT_2_C High   'MainMol' Ueq as Compared to Neighbors of        C97 Check
PLAT241_ALERT_2_C High   'MainMol' Ueq as Compared to Neighbors of       C107 Check
PLAT242_ALERT_2_C Low    'MainMol' Ueq as Compared to Neighbors of        C61 Check
PLAT250_ALERT_2_C Large U3/U1 Ratio for Average U(i,j) Tensor ....        2.1 Note
PLAT260_ALERT_2_C Large Average Ueq of Residue Including       N62      0.122 Check
PLAT260_ALERT_2_C Large Average Ueq of Residue Including       N84      0.121 Check
PLAT341_ALERT_3_C Low Bond Precision on  C-C Bonds ..............    0.01032 Ang.
```

Alert level G

```
PLAT007_ALERT_5_G Number of Unrefined Donor-H Atoms ..............          6 Report
PLAT012_ALERT_1_G N.O.K.   _shelx_res_checksum Found in CIF ......     Please Check
PLAT045_ALERT_1_G Calculated and Reported Z Differ by a Factor ...          2 Check
PLAT083_ALERT_2_G SHELXL Second Parameter in WGHT  Unusually Large      66.68 Why ?
PLAT794_ALERT_5_G Tentative Bond Valency for Mn1       (II)      .       2.17 Info
PLAT794_ALERT_5_G Tentative Bond Valency for Mn2       (II)      .       2.15 Info
PLAT883_ALERT_1_G No Info/Value for _atom_sites_solution_primary .     Please Do !
PLAT965_ALERT_2_G The SHELXL WEIGHT Optimisation has not Converged     Please Check
```

1 **ALERT level A** = Most likely a serious problem - resolve or explain
4 **ALERT level B** = A potentially serious problem, consider carefully
17 **ALERT level C** = Check. Ensure it is not caused by an omission or oversight
8 **ALERT level G** = General information/check it is not something unexpected

5 ALERT type 1 CIF construction/syntax error, inconsistent or missing data

```
15 ALERT type 2 Indicator that the structure model may be wrong or deficient
 4 ALERT type 3 Indicator that the structure quality may be low
 3 ALERT type 4 Improvement, methodology, query or suggestion
 3 ALERT type 5 Informative message, check
```

It is advisable to attempt to resolve as many as possible of the alerts in all categories. Often the minor alerts point to easily fixed oversights, errors and omissions in your CIF or refinement strategy, so attention to these fine details can be worthwhile. In order to resolve some of the more serious problems it may be necessary to carry out additional measurements or structure refinements. However, the purpose of your study may justify the reported deviations and the more serious of these should normally be commented upon in the discussion or experimental section of a paper or in the "special_details" fields of the CIF. checkCIF was carefully designed to identify outliers and unusual parameters, but every test has its limitations and alerts that are not important in a particular case may appear. Conversely, the absence of alerts does not guarantee there are no aspects of the results needing attention. It is up to the individual to critically assess their own results and, if necessary, seek expert advice.

Publication of your CIF in IUCr journals

A basic structural check has been run on your CIF. These basic checks will be run on all CIFs submitted for publication in IUCr journals (*Acta Crystallographica*, *Journal of Applied Crystallography*, *Journal of Synchrotron Radiation*); however, if you intend to submit to *Acta Crystallographica Section C* or *E* or *IUCrData*, you should make sure that full publication checks are run on the final version of your CIF prior to submission.

Publication of your CIF in other journals

Please refer to the *Notes for Authors* of the relevant journal for any special instructions relating to CIF submission.

PLATON version of 10/05/2023; check.def file version of 10/05/2023

Datablock 5 - ellipsoid plot

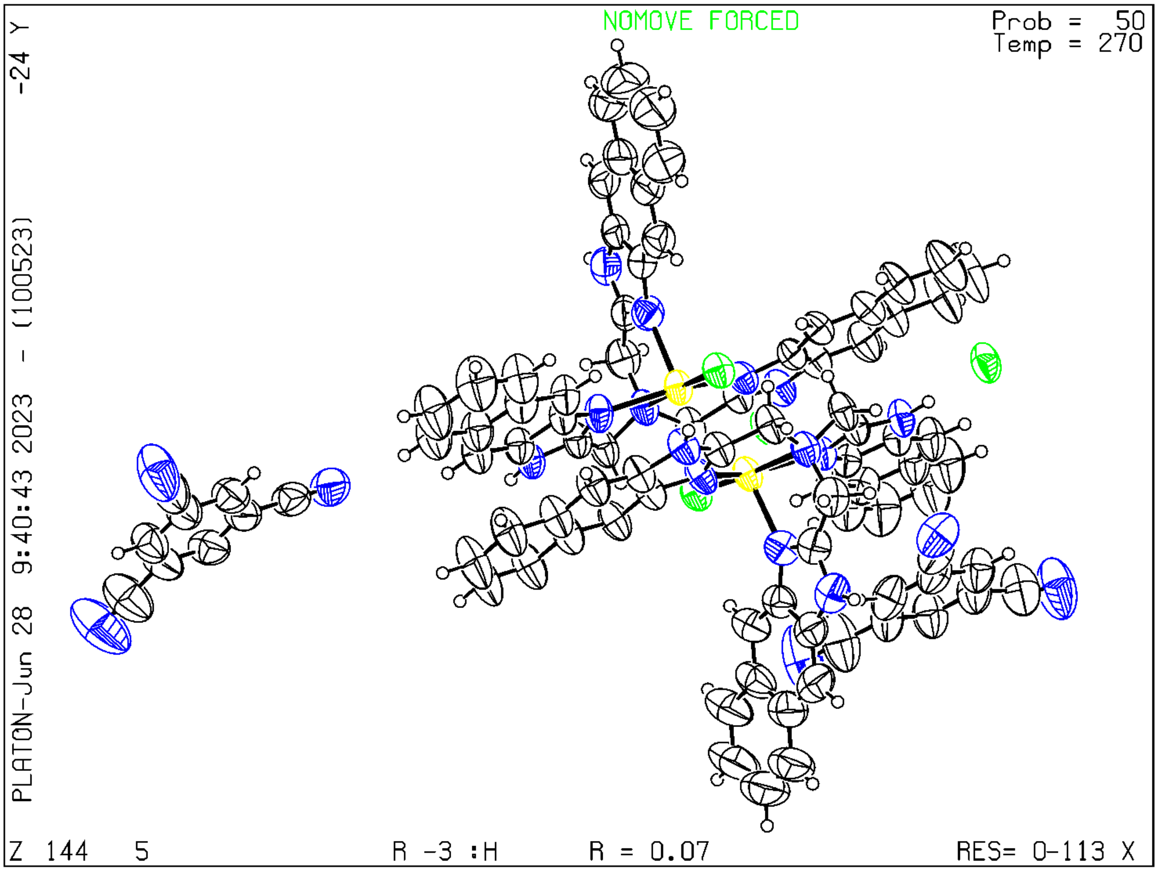

Supplement: Supplementary file 2 — Supporting Information [file ADVS-11-2308028-s001.zip › Check-Cif-Activated 1-270 K.pdf]
